# Pregnant Women in Louisiana Are Not Meeting Dietary Seafood Recommendations

**DOI:** 10.1155/2016/1853935

**Published:** 2016-07-18

**Authors:** M. L. Drewery, A. V. Gaitán, C. Thaxton, W. Xu, C. J. Lammi-Keefe

**Affiliations:** ^1^Louisiana State University, Baton Rouge, LA 70803, USA; ^2^Louisiana State University AgCenter, Baton Rouge, LA 70803, USA

## Abstract

*Background*. The 2015–2020 Dietary Guidelines for Americans recommend that pregnant women and women of childbearing ages consume 8–12 oz. of seafood per week. Fish are the major dietary source of omega-3 long chain polyunsaturated fatty acids, which have benefits for the mother and fetus.* Methods*. In this observational study, we investigated dietary habits of pregnant women in Baton Rouge, Louisiana, USA, to determine if they achieve recommended seafood intake. A print survey, which included commonly consumed foods from protein sources (beef, chicken, pork, and fish), was completed by pregnant women at a single-day hospital convention for expecting families in October 2015. Women (*n* = 221) chose from six predefined responses to answer how frequently they were consuming each food.* Results*. Chicken was consumed most frequently (75% of women), followed by beef (71%), pork (65%), and fish (22%), respectively. Consumption frequency for the most consumed fish (catfish, once per month) was similar to or lower than that of the least consumed beef, chicken, and pork foods. Consumption frequency for the most consumed chicken and beef foods was at least once per week.* Conclusion*. Our data indicate that pregnant women in Louisiana often consume protein sources other than fish and likely fail to meet dietary seafood recommendations.

## 1. Introduction

Optimal fetal development and infant outcome depend on availability of specific nutrients during the preconceptual and gestational periods, including the omega-3 long chain polyunsaturated fatty acids (LCPUFAs), docosahexaenoic acid (DHA), and eicosapentaenoic acid (EPA) [1, 2].

In response to maternal omega-3 LCPUFA intake during pregnancy, infants have improved performance on cognitive and developmental tests [3–5], accelerated maturation of the visual and autonomic nervous systems [6–8], and leaner body composition [9, 10].

Health benefits of omega-3 LCPUFA intake in pregnancy may also extend to the mother. The relationship between dietary omega-3 LCPUFA intake and maternal mental health conditions (depressive disorders during and after pregnancy) has been examined. There is evidence that omega-3 LCPUFA intake may benefit women with preexisting depressive illnesses [11–14]. These findings are complemented by observational studies which point to an association between low dietary omega-3 LCPUFA intake, especially DHA, and increased risk of depressive disorders during and following pregnancy [15, 16].

There is evidence that omega-3 LCPUFAs also positively affect general pregnancy outcome. Omega-3 LCPUFAs prolong pregnancy duration, reducing the risk of birth before 34 gestational weeks by 31% in normal and 61% in high-risk pregnancies [17, 18]. Increasing pregnancy duration has implications for decreasing incidence of preterm birth and intrauterine growth retardation [19].

These measurable and documented benefits of omega-3 LCPUFA underscore the recommendations of the 2015–2020 Dietary Guidelines for Americans [20] that pregnant women and women of childbearing ages consume 8–12 oz., or two to three 4 oz. servings, of seafood per week, as cold water marine fish are the major dietary source of omega-3 LCPUFAs (Table 1) [21]. In general, fish are regarded as good dietary sources of omega-3 LCPUFAs; however, fatty acid content depends on variety, geographical location, method of farming/harvesting, and other factors [21].

These recommendations translate to approximately 250 mg omega-3 LCPUFAs per day and are in line with the recommendation of 200 mg DHA per day set forth by an international panel of experts in an earlier consensus statement [22].

The Food and Drug Administration and Environmental Protection Agency further specify that servings should be from a variety of fish that have low levels of methylmercury [23]. Nearly all fish contain trace amounts of methylmercury; however, larger fish with longer lifespans have greater accumulations of the neurotoxin [24]. As methylmercury crosses the placenta, it accumulates in the fetus at higher concentrations than those in the mother [25, 26]. Fetal exposure to excess amounts of methylmercury in utero, when the brain is especially vulnerable to environmental insults, can negatively affect brain and nervous system development [27]. Tilefish from the Gulf of Mexico, shark, swordfish, and king mackerel contain high levels of methylmercury [21, 23]; thus, pregnant women and women of childbearing ages are advised to avoid these fish [23].

During pregnancy, the fetus relies on maternal intake and placental transfer of nutrients to meet developmental demands. Although prenatal vitamins and other vitamins/supplements are marketed to pregnant women, they may not contain omega-3 LCPUFAs or women may not consume them at all or with any regularity [28]. Thus, low fish intake during pregnancy could result in low fetal accumulation of DHA and EPA.

Previous estimates of dietary omega-3 LCPUFA intake point to low consumption by pregnant women and women of childbearing ages. In a small sample (*n* = 21) of pregnant women in Baton Rouge, Louisiana, USA, data from our laboratory [28] indicate the average dietary intake of DHA is 72 mg per day, which translates to 95% of pregnant women not meeting the recommendation of 200 mg DHA per day [22]. When supplement intake was taken into consideration, 62% of pregnant women still failed to meet the recommended DHA intake. In an earlier study, we reported that nonpregnant women of childbearing ages (*n* = 183; average age: 20 years; age range: 18–28 years) consumed an average of 66 mg DHA per day, which included both dietary and supplemental sources of DHA [29].

Given the role of omega-3 LCPUFAs in infant development, pregnancy outcome, and maternal health, it is important to assess if pregnant women are adhering to the dietary recommendation to include seafood in their diets and, if not, what foods they are choosing to consume instead. Therefore, the aim of the study was to investigate the dietary habits of pregnant women in Baton Rouge, Louisiana, USA. Specifically, we evaluated their consumption of various dietary protein sources.

Geographically, Baton Rouge is located directly on the Mississippi River and approximately 157 miles north of the Gulf of Mexico. As Louisiana is a coastal state and fish are an intricate part of the regional culture and cuisine [30], we hypothesized that pregnant women in the Greater Baton Rouge area would meet the recommended seafood intake for pregnant women and women of childbearing ages.

## 2. Materials and Methods

### 2.1. Study Overview

For this observational study, we approached women at an event held for expecting women and their partners at a hospital in Baton Rouge, Louisiana. The free, single-day event was held in October 2015. Women were approached and invited to complete a survey about their dietary habits during pregnancy and respond to a demographic questionnaire; the survey and questionnaire were provided as separate documents. All pregnant women who visited the research booth at the event were invited to participate; the only inclusion criterion was current pregnancy and there was no selection bias. Our efforts resulted in 221 completed surveys and questionnaires; the responses from each were separated at the time of completion.

Compensation for study completion was entry into a raffle for free baby books and other materials for expecting families. Women provided their first name and telephone number on a separate piece of paper; this paper was not attached to the survey or questionnaire. When a name was drawn in the raffle, the woman was contacted by a call or text and returned to the booth to pick up her raffle prize. All contact information was destroyed at the conclusion of the convention.

The survey included a statement that completion of the survey constituted consent to participate and participation were voluntary. All procedures involving human subjects were approved by The Louisiana State University AgCenter and Woman's Hospital Institutional Review Boards.

Contact and demographic information were not attached to the survey and, as such, responses were anonymous. The women were allowed to complete their survey and the questionnaire and provide their contact information on an individual clipboard standing away from the table at which the researchers were stationed.

### 2.2. Survey and Demographic Questionnaire

The survey was designed to be completed by participants in approximately 5 minutes with minimal input or direction from the researchers. Women were instructed to complete the survey in accordance with their usual dietary habits during pregnancy. The survey has not been previously validated and was developed as a tool to provide preliminary, descriptive data that provides a direction and foundation upon which to build for future research.

The survey contained four sections (“protein sources”), labeled and ordered as follows: “Beef,” “Chicken,” “Fish,” and “Pork.” Each section included a list of foods commonly consumed for that respective protein source; these foods were subjectively chosen by the researchers.

“Beef” included, in order, “Steak,” “Hamburger,” “Stew meat,” “Brisket,” and “Roast.” “Chicken” included, in order, “Wings,” “Breast,” and “Legs.” “Fish” included, in order, “Canned tuna,” “Tuna steak,” “Tilapia,” “Salmon,” “Cod,” “Catfish,” “Swordfish,” “Trout,” “Bass,” “Flounder,” and “Herring.” “Pork” included, in order, “Chop,” “Tenderloin,” and “Roast.”

More specific information about the foods and food preparation was not sought. For example, “Steak” could include any cut of steak, “Salmon” could include any species of salmon, and “Wings” could include any preparation and/or cooking style of chicken wings.

Each question had six predefined responses to assess how frequently the women were consuming each: “Never,” “Once/week,” “2-2+/week,” “Once/month,” “2-3/month,” and “4-4+/month.” The majority of women checked only one box per food; however, if multiple or none of the boxes were checked, that data point was entered as missing.

As the primary focus of our study was fish consumption by pregnant women, we constructed our survey to include a variety of fish, including those that are poor and good sources of omega-3 LCPUFAs and those that are indigenous and nonnative to the area (canned tuna, tuna steak, tilapia, salmon, cod, catfish, swordfish, trout, bass, flounder, and herring).

The demographic questionnaire, included as a separate document, included questions about participant age, ethnicity, education level, and if she was a first-time mother. All documents were provided in print.

### 2.3. Interpretation of Results

Our survey did not indicate the size of a serving. Rather, we asked how often the women consumed each food and assumed portion sizes for each. In speculating whether pregnant women are meeting the omega-3 LCPUFA recommendations by dietary fish intake, we assumed each serving to be 4 oz.

This assumption was based on a table in the 2015–2020 Dietary Guidelines for Americans [20]. Nutritional aspects of common seafood varieties were provided for 4 oz. portions of each. Although the guidelines specify that pregnant women should “consume 8 to 12 oz. of seafood per week from a variety of sources”, a serving size is not defined. However, The Food and Drug Administration and Environmental Protection Agency specify that the recommended 8–12 oz. translates to two to three servings of fish per week [23].

## 3. Results 

### 3.1. Population Demographics

Demographic data (*n* = 221) for our survey population are provided in Table 2. The majority of the women in our population were Caucasian (71%), 26–30 years old (37%), and had completed some college (24%), a 4-year college degree (29%), or a graduate degree (29%). African American was the second most common ethnicity (20%) and 20–25 years of age was the second most common age range (29%). First-time mothers comprised the majority of our population (79%).

### 3.2. Response Rate

Of the women approached (estimated 250–275), 221 completed the survey. The average response rate for each food was 92%. Women responded to the frequency with which they ate stew meat least often (i.e., did not answer the question; 88% response rate) and chicken breast most often (96% response rate).

### 3.3. Consumption Habits of Pregnant Women: Fish

**Figure 1:**
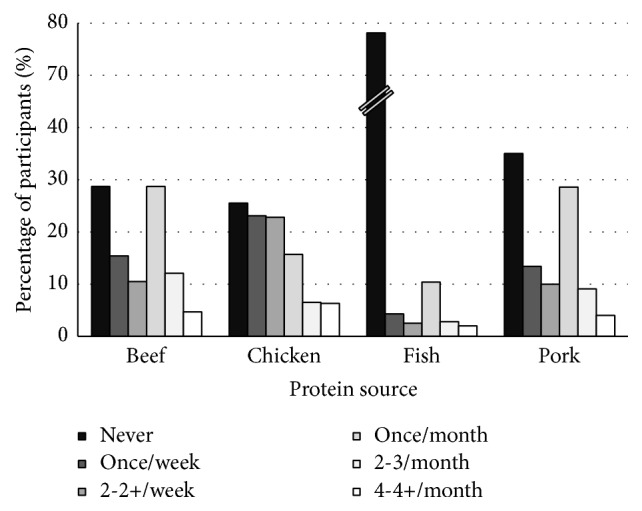
Consumption rate and frequency of protein sources by pregnant women.

Twenty-two percent of women reported consuming fish, when consumption of all individual varieties was averaged (Figure 1). Catfish was consumed by a majority of the population, 59% of women. Tilapia, canned tuna, and salmon were consumed by 47, 40, and 35% of women, respectively. Swordfish, herring, and flounder were consumed by less than 3% of women.

**Figure 2:**
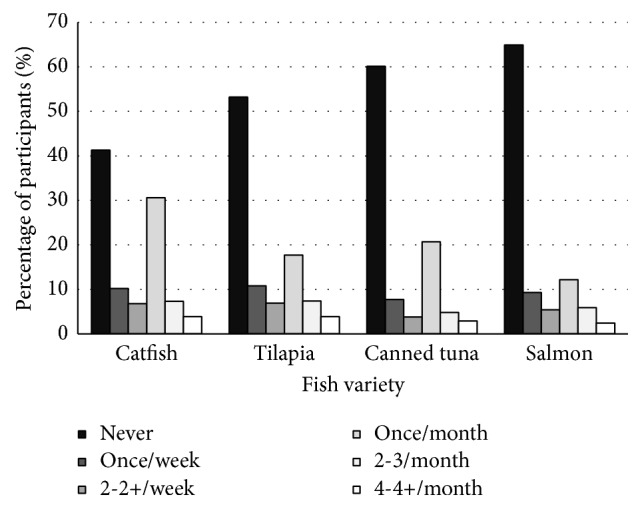
Consumption rate and frequency of the more consumed fish varieties by pregnant women.

**Figure 3:**
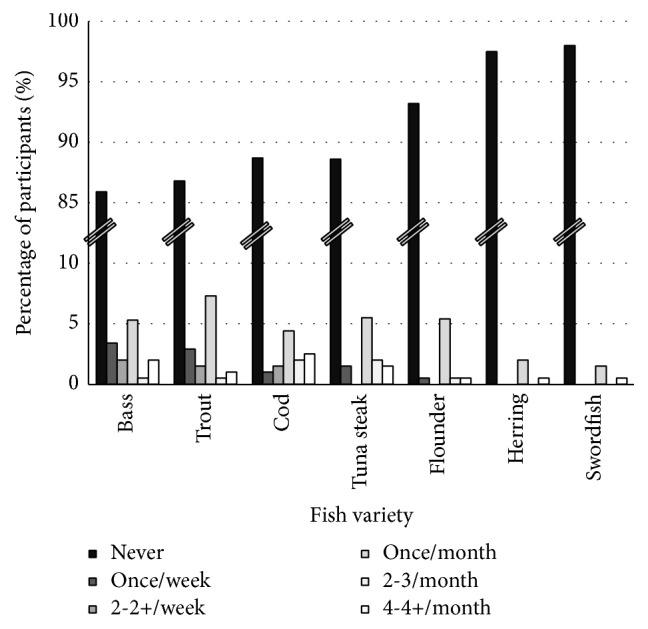
Consumption rate and frequency of the less consumed fish varieties by pregnant women.

The most common consumption frequency for catfish, tilapia, canned tuna, and salmon was once per month, followed by once per week. Consumption rate and frequency for each fish variety are presented in Figures 2 and 3.

### 3.4. Consumption Habits of Pregnant Women: Beef, Chicken, and Pork

Consumption rate for beef, chicken, and pork, when all foods were averaged within protein source, was 71, 74, and 65%, respectively. Hamburger, chicken breast, and pork chops were the most consumed foods for each protein source, with 90, 92, and 63% of women reporting that they consume each, respectively. Brisket, chicken legs, and pork roast were the least consumed foods for each protein source, with 52, 65, and 58% of women reporting that they consume each, respectively.

**Figure 4:**
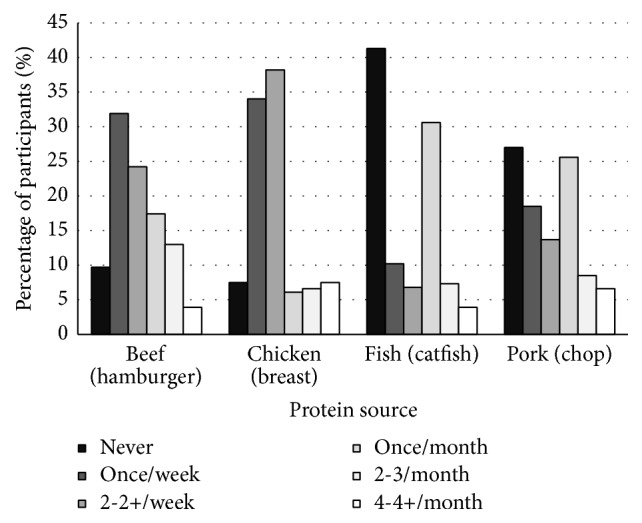
Consumption rate and frequency of the most consumed foods for each protein source by pregnant women.

**Figure 5:**
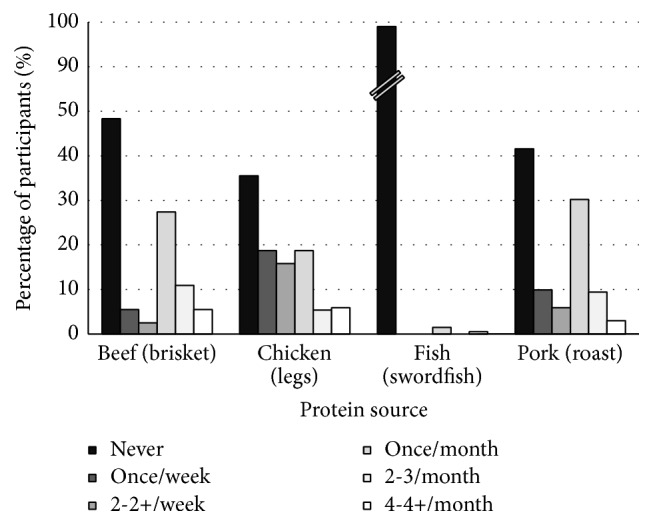
Consumption rate and frequency of the least consumed foods for each protein source by pregnant women.

Women most commonly reported intake of the most consumed beef (hamburger) and chicken foods (chicken breast) at a frequency of once or at least twice per week. A consumption frequency of once per month was the most common response for the least popular beef, chicken, and pork foods (brisket, chicken legs, and pork roast, resp.). The most popular pork food (pork chops) was most often consumed at a frequency of once per month. Consumption rate and frequency of consumption for the most and least consumed foods, grouped by protein source, are presented in Figures 4 and 5, respectively.

## 4. Discussion

### 4.1. Achieving Dietary Omega-3 LCPUFA Recommendations for Pregnant Women

The two most commonly consumed fish varieties by our population (catfish and tilapia) have significantly lower concentrations of omega-3 LCPUFAs than the varieties which were rarely consumed (Table 1) [21]. Of particular interest is the finding that canned tuna, which is widely available, is inexpensive, has a long shelf life, and is amenable to easy preparation [31], was not consumed by more women or more frequently. Salmon, a similar variety to canned tuna in terms of preparation and favorable omega-3 LCPUFA content [21], was also consumed at a low frequency.

To meet the recommendation of an average intake of 250 mg omega-3 LCPUFA per day [20], one would have to consume 12 oz. of farmed catfish or 9.7 oz. of tilapia every day (Table 1) [21]. This equates to approximately 21 servings of farmed catfish or 17 servings of tilapia per week, assuming a 4 oz. serving size. As the women in our population reported consuming catfish, tilapia, and canned tuna (the three most consumed varieties) each at a frequency of once per month, they are likely to not be achieving recommended intakes of omega-3 LCPUFAs.

Dietary incorporation of canned white tuna and/or salmon at a frequency of twice per week would satisfy recommended levels of omega-3 LCPUFA intake, exclusive of intake of other varieties (Table 1) [21].

### 4.2. Intake of Fish Known to Have High Methylmercury Content

The Food and Drug Administration and Environmental Protection Agency advise pregnant women and women of childbearing ages to avoid consumption of tilefish from the Gulf of Mexico, shark, swordfish, and king mackerel due to their high methylmercury content [23]. In our population, three women (1.4%) reported consumption of swordfish at a frequency of once per month and one (0.5%) indicated she consumed swordfish at least four times per month. The other varieties were not included in our survey. These data may point to a need to further emphasize the recommendation that pregnant women and women of childbearing ages should avoid fish known to contain high levels of methylmercury [23].

### 4.3. Comparison of Dietary Intake of Fish and Other Protein Sources

In the current study, consumption rate of beef, chicken, and pork was at least threefold higher than that of fish. The most consumed fish varieties were consumed at a frequency that was similar to or less than that of the least consumed beef, chicken, and pork foods. Clearly, when choosing a protein source, pregnant women are opting to consume beef, chicken, and/or pork in favor of fish.

### 4.4. Comparison with Previous Findings and International Differences in Dietary Seafood Habits

Our data is in line with that of a previous investigation [32] in which it was reported that 89% of pregnant women in Massachusetts, USA, consume less than 3 fish meals per month and the average canned tuna consumption is 2 servings per month. Similarly, pregnant women in Ontario, Canada [33], were reported to consume 0.7 fish meals per week, which equates to less than 3 fish meals per month. These findings are also similar to those for pregnant women in southwestern Quebec, Canada, where the women consumed 3.6 fish meals per month [34].

There is a stark difference in dietary seafood habits of pregnant women between the North American countries of the United States and Canada and that of other regions.

A large, observational study [35] found that 88% of pregnant women in the United Kingdom consumed at least 8 seafood meals per month. Pregnant Norwegian women, on average, consumed approximately 45 seafood meals per month [36] and 77% of pregnant women living on the Faroe Islands consumed at least 12 seafood meals per month [37].

A comparison of two studies assessing pregnant women in Denmark [38] and Netherlands [39] revealed that 22% of the Danish population consumed at least 560 g of fish per month (equivalent to 4.9 servings) versus 56% of the Dutch population. These results are in agreement with those in a different Danish population [40], where the average fish consumption for pregnant women was 3.9 meals per month. Pregnant Swedish women were reported to consume 6.7 fish/shellfish meals per month, with less than 1% of women reporting they never consumed fish at all [41].

In Spanish populations, 86% of pregnant women reported consuming at least 12 seafood meals per months [42] and 61% of pregnant women ate canned tuna at a minimal frequency of 4 times per month [43]. Findings from a Taiwanese study indicate 99% of pregnant women in Taipei consumed fish during pregnancy at an average rate of 11 meals per month [44].

Although those North American populations outlined above [32–34], along with the population in the current study, all live within 160 miles of the coast or a major body of water, it is apparent that pregnant women in these locations eat less seafood than their European and East Asian counterparts. The international difference in fish and seafood consumption is likely fueled by the typical Western diet that is characteristic of North America, as supported by our current observation that pregnant women in Baton Rouge, Louisiana, USA, have a strong preference for hamburger or chicken breast.

Given the wide availability of seafood in coastal regions [45], one may expect pregnant women living in inland locations in North America to have even lower seafood intakes than those reported in the current and previous studies. It is important to note, as well, that our population is well educated, with 90% having completed some college. Thus, even pregnant women who are educated are not consuming recommended amounts of fish.

The international disparity in seafood intake reflects the findings of a 2010 study, which qualitatively determined knowledge and behavior of pregnant women (*n* = 22) in Northeastern USA with regard to fish consumption [46]. The researchers found that while a fair amount of pregnant women (46%) was aware that fish contained a potential toxic contaminant (methylmercury), less knew that fish contained DHA (36%) or a function of DHA during pregnancy (23%). Furthermore, none of the women (0%) had been advised to consume fish during pregnancy.

Two studies [32, 34] from 2003 and 2004 found that women from Northeastern USA and Canada more often maintained or reduced fish intake after becoming pregnant rather than increasing it. The decreased consumption after becoming pregnant was calculated to be 1.4 servings per month [34]. The authors speculated that this effect was a result of national mercury advisories in the early 2000s which recommended pregnant women limit consumption of certain fish [32]. These findings contrast those of the aforementioned study in Taiwan [44], conducted in 2006, where the percentage of women who consumed fish increased from 95 to 99% upon becoming pregnant. Thus, our data may suggest that, in 2015, pregnant women in North America may remain uncomfortable incorporating fish as a dietary protein source and opt for chicken, beef, or pork foods instead.

It is important to note that, for each study outlined above, dietary data were collected from and reported in a variety of ways. For comparison with our results, we converted the data to servings per month by assuming a serving was 4 oz., if the data were reported as g consumed per unit of time. We note that dietary data were collected by various methods (food frequency questionnaires, 24-hour dietary recalls) but assumed each method to be equal. These data manipulations could affect the precision of our comparisons.

### 4.5. Future Research Direction

Future studies should assess whether pregnant women and women of childbearing ages have knowledge of the dietary recommendations for seafood consumption. These efforts should aim to elucidate if (1) there are specific groups of pregnant women who are less likely to meet dietary fish recommendations and (2) why these women fail to meet those recommendations.

Replication of the current survey across different geographic areas would also provide insight into the effect of coastal versus inland location on fish intake and dietary protein preferences.

### 4.6. Study Limitations

Our survey was conducted in a convenience sample and since the survey and demographic questionnaires were not connected, we are unable to examine potential group differences or correlations between demographic parameters and responses.

We assumed values for portion sizes. Although this assumption does not affect our observations of dietary habits, it does affect the precision of our calculations in regard to whether pregnant women are meeting omega-3 LCPUFA recommendations or not. Furthermore, we did not consider how foods were prepared. Certain cooking styles are related to differences in the fatty acid content of the resulting product [21]. This, too, affects calculations of omega-3 LCPUFA intake.

The characteristics of our study population differ from those published by the United States Census Bureau [47] for Baton Rouge, Louisiana. The population of Baton Rouge are African Americans (55%) or Caucasians (39%) who have completed high school (26%) or some college (23%). The overall population of Louisiana is more similar to that of our study population; 63% Caucasian or 32% African Americans who have completed high school (26%), some college (23%), or a 4-year college degree (19%).

It is important to note that educational attainment data from the United States Census Bureau data reflects that of the population aged 25 years and older, without specificity to gender. Approximately 32% of our population was aged 25 years or less. Additionally, the ethnic breakdowns provided by the United States Census Bureau data reflect that of the entire population in that region without regard to age, gender, or pregnancy status. These discrepancies make it difficult to draw conclusions on the generalizability of our data.

## 5. Conclusion

These data reveal that pregnant women in Baton Rouge, Louisiana, USA, are not meeting dietary recommendations for seafood consumption and, therefore, likely do not consume adequate amounts of omega-3 LCPUFAs for optimal maternal health, fetal development, and infant outcome. These data also reveal the protein sources and specific foods that pregnant women are consuming in lieu of fish.

The apparent deficit in omega-3 LCPUFA intake has major implications during and after pregnancy and should be addressed with intensified efforts to provide nutrition and lifestyle education to pregnant women and women of childbearing ages.

Although our data indicate pregnant women, in general, do not meet dietary seafood recommendations, future research will help us better understand the habits of pregnant women, directing us in our development of targeted education efforts which emphasize the importance of consumption of fish low in methylmercury during pregnancy.

## Figures and Tables

**Table 1:** DHA and EPA content of major dietary sources of omega-3 LCPUFA^1,2^.

	DHA, mg/4 oz.	EPA, mg/4 oz.	Number of 4 oz. servings to provide 250 mg DHA + EPA^3^	Oz. to provide 250 mg DHA + EPA
Bass				
Sea	492	183	0.37	1.48
Striped	663	192	0.29	1.17
Catfish				
Farmed	64	19	3.02	12.10
Wild	265	147	0.61	2.43
Cod				
Atlantic	136	72	1.20	4.81
Pacific	109	39	1.69	6.76
Herring				
Atlantic	977	804	0.14	0.56
Pacific	781	1099	0.13	0.53
Flounder	123	155	0.90	3.61
Salmon				
Atlantic, farmed	1251	977	0.11	0.45
Atlantic, wild	1264	364	0.15	0.61
Pink	377	207	0.43	1.71
Sockeye	1797	395	0.11	0.46
Tilapia	97	5	2.44	9.74
Trout	599	229	0.30	1.21
Tuna				
Bluefin	1009	321	0.19	0.75
Light, canned in water	223	32	0.98	3.93
Yellowfin	100	13	2.21	8.82
White, canned in water	713	264	0.26	1.02

^1^Adapted from the USDA National Nutrient Database for Standard Reference, Release 27 [21]; DHA: docosahexaenoic acid; EPA: eicosapentaenoic acid; LCPUFA: long chain polyunsaturated fatty acid.

^2^Nutrient values are estimates and depend on species of fish, total fat content of fish, geographical location, method of raising/harvesting, and cooking. All values are for raw portions and, as such, are overestimates after cooking is considered [21].

^3^Number of servings (4 oz.) were calculated to meet 250 mg of omega-3 LCPUFA per day, as recommended for pregnant women by the Dietary Guidelines for Americans (2015–2020) [20].

**Table 2:** Demographics of the survey population.

	% of women, *n* = 221
Age, years	
<20	3.2
20–25	29.0
26–30	37.3
31–35	23.0
36–40	6.9
No answer	0.5

Education	
Some high school	3.2
High school graduate	6.5
Some college	23.5
2-year degree	8.8
4-year degree	29.0
Graduate degree	28.6
No answer	0.5

Ethnicity	
African American	20.3
Caucasian	71.4
Hispanic	2.3
American Indian	0.5
Asian	4.6
Multiracial	0.5
No answer	0.5

First-time mom	
Yes	78.5
No	21.5
No answer	1.4
